# A giant fourth-ventricular tuberculoma mimicking a primary posterior fossa tumor

**DOI:** 10.2478/abm-2021-0024

**Published:** 2021-08-20

**Authors:** Minh Duc Nguyen

**Affiliations:** Department of Radiology, Hanoi Medical University, Hanoi 100000, Vietnam; Department of Radiology, Pham Ngoc Thach University of Medicine, Ho Chi Minh City 700000, Vietnam; Department of Radiology, Children's Hospital 02, Ho Chi Minh City 700000, Vietnam

**Keywords:** fourth ventricle, infratentorial neoplasms, tuberculoma, intracranial, tuberculosis

## Abstract

Typically, an intracranial tuberculoma occurs within the brain parenchyma. Intraventricular tuberculomas are rare in the absence of systemic tuberculosis (TB), and the differential diagnosis between tuberculoma and other lesions, such as primary brain tumors, can be difficult. We report an extremely unusual case of solitary fourth-ventricular tuberculoma, which occurred in a 3-year-old female patient, with no indication of TB. This lesion appeared as a primary intraventricular tumor in the fourth ventricle in both clinical and radiological examinations. In this scenario, a surgical treatment option was pursued. Histopathological testing supported the diagnosis of tuberculoma. The patient was subsequently treated with 18 months’ therapy for tuberculous, without adverse events.

Tuberculosis (TB) is a widespread infectious disease caused by *Mycobacterium tuberculosis* that endures as a public health concern in developing countries [1]. TB most commonly presents as a pulmonary infection, but might affect other organs and is generally transmitted through droplet nuclei generated by patients with active lung TB [2]. Extrapulmonary TB refers to a pathological condition, caused concurrently with TB, which affects organs other than the lungs, such as the pleura, genitourinary tract, lymphatic nodes, spine, bones, joints, or meninges [3]. Central nervous system (CNS) TB adversely impacts about 1% of all patients infected with *M. tuberculosis*, and might present as a tuberculous abscess, tubercular meningitis, or a tuberculoma [4].

A tuberculoma is a tumor-like presentation, often positioned in the cerebellar and cerebral parenchyma [4, 5]. The ventricles in the brain are typically protected by strong immune responses and vascular barriers that shield them against tuberculous infection; hence, tuberculoma is quite uncommon in the ventricular system [6]. Additionally, the absence of ventricular tuberculoma-specific clinical symptoms renders it extremely difficult to establish a precise diagnosis between ventricular tuberculoma and primary CNS tumors [6,7,8,9,10,11,12,13,14].

This article reports a very unusual case of tuberculoma, without pulmonary TB, which mimicked a primary predominant tumor in the fourth ventricle and describes the difficulties encountered during diagnosis and treatment.

## Case presentation

**Figure 1:**
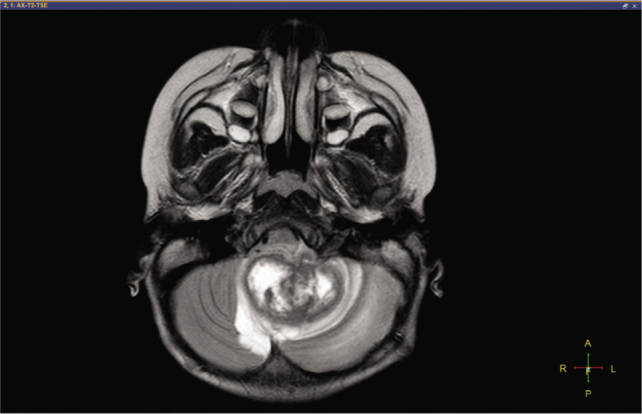
Axial T2-weighted image shows a predominantly cystic mass located in the fourth ventricle.

**Figure 2:**
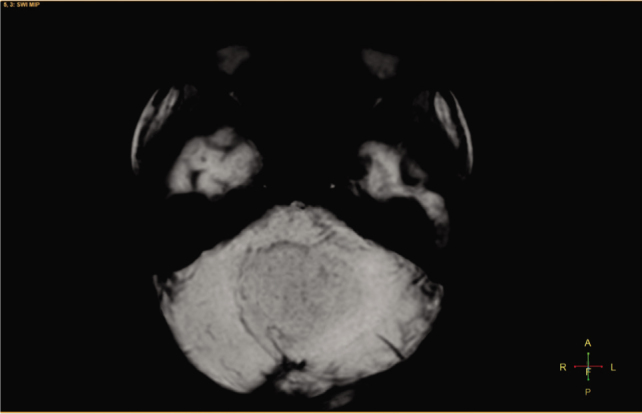
Axial susceptibility-weighted image shows no calcification or hemosiderin inside the mass.

**Figure 3:**
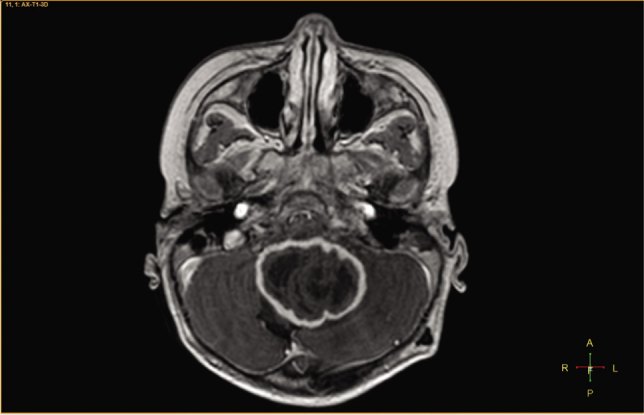
Axial T1-weighted image with contrast agent reveals a giant ring-enhancing mass.

**Figure 4:**
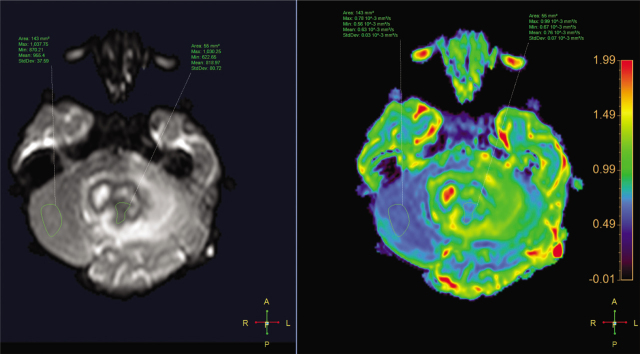
Axial diffusion-weighted image (left panel) and apparent diffusion coefficient (ADC) map (right panel) of the intraventricular lesion (region of interest (ROI) central) and normal-appearing parenchyma (ROI left).

**Figure 5:**
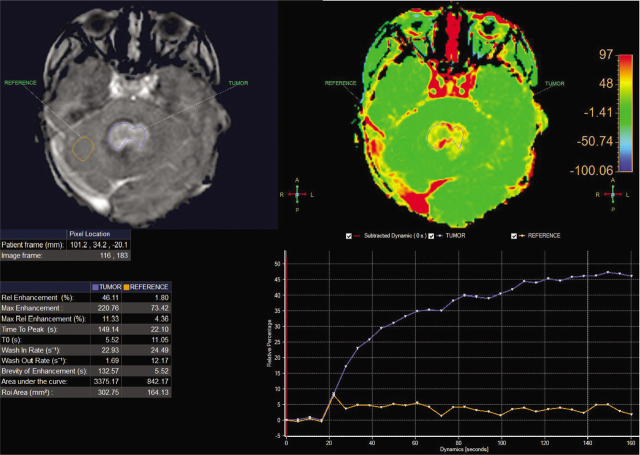
Axial T1-perfusion image (upper left) and perfusion map (upper right) of the lesion (tumor) and normal-appearing parenchyma (reference). T1-perfusion parameters (lower left); and time–signal intensity curves for the tumor (blue) and the parenchyma (orange) (lower right).

A 3-year-old girl was admitted to Children's Hospital 2, Ho Chi Minh city, Vietnam, with a 3-month history of severe headache, nausea, and vomiting. The patient's medical profile was unremarkable, without TB risks or immunocompromising diseases. The patient received a Bacillus Calmette–Guérin (BCG) vaccination after birth and she had no history of lymphadenopathy. Immunodeficiency disorders were not detected. Her neurological examination was unremarkable, without focal neurological deficits. No signs of infection and no abnormalities were detected on a chest radiograph. The patient underwent 1.5 tesla magnetic resonance imaging (MRI) of the brain, with a gadolinium-based contrast agent (Gadovist; Bayer) at a dose of 0.1 mmol per kilogram body weight. The axial T2-weighted image showed a large intra-fourth-ventricular, central hyperintensity with a peripheral thick irregular T2 hypointensity mass, which was clearly defined by surrounding vasogenic edema (**Figure 1**). The right-to-left (RL), anterior-to-posterior (AP), and feet-to-head (FH) diameters of the mass were 42 mm, 34 mm, and 31 mm, respectively. No ossification or hemorrhage was detected within the mass on susceptibility-weighted imaging (**Figure 2**). The mass was typically ring-enhancing, with central necrosis, on T1-weighted image using contrast enhancement (**Figure 3**). The mean apparent diffusion coefficients for the solid component of the mass and the normal-appearing parenchyma were 0.76 × 10^−3^ mm^2^/s and 0.63 × 10^−3^ mm^2^/s, respectively (**Figure 4**). On the T1-perfusion map, the relative enhancement (%), maximum enhancement, maximum relative enhancement (%), time to peak (s), wash-in rate (s^−1^), wash-out rate (s^−1^), and area under the curve values for the mass compared with the normal-appearing parenchyma were as 46.11 vs 1.80, 220.76 vs 73.42, 11.33 vs 4.36, 149.14 vs 22.10, 22.93 vs 24.49, 1.69 vs 12.17, and 3375.17 vs 842.17, respectively (**Figure 5**). The clinical and radiological information suggested the initial diagnosis, in this case, of a suspiciously anaplastic ependymoma or a high-grade glioma. Neuroradiologists and neurosurgeons fully agreed to recommend surgery as the optimal treatment option. Her preoperative human immunodeficiency virus (HIV) test was negative. The patient underwent radical tumor eradication surgery, and the histopathological findings revealed granulomatous inflammation, coupled with caseous necrosis inside the lesion, consistent with a tuberculoma. Hematoxylin and eosin staining showed epithelioid histocytes and Langhans giant cells surrounding the necrotic areas. Acid-fast bacilli (AFB) were noted in the tissue sample. Additionally, polymerase chain reaction (PCR) for *M. tuberculosis* was performed and its result was positive. The postoperative period was uneventful. Antituberculous therapy including isoniazid, rifampicin, pyrazinamide, and ethambutol was administered for 18 months after surgery. This report has been approved by the hospital ethics committee (Ref: 352/NĐ2-CĐT) and the patient's anonymity has been preserved as far as possible. The mother of the patient has provided explicit documented consent for publication of this report and accompanying images and has been provided the opportunity to see and read the material submitted for publication.

## Discussion

Epidemiologically, 15% of all TB cases are extrapulmonary TB, without lung involvement [15]. CNS TB comprises only 1% of all TB cases, but is associated with the worst prognosis [16]. Tuberculomas are the most prevalent tuberculous lesions, along with leptomeningitis. In endemic nations, they are responsible for 10%–30% of expanding intracranial processes. The incidence of tuberculomas in tuberculous meningitis is believed to be between 4% and 28%. Unlike in adults, pediatric tuberculoma with intraventricular localization is extremely rare, with only 5 cases involving pediatric patients (tuberculomas related to the lateral ventricle, third ventricle, and thalamus) reported to our knowledge, and no cases of intra-fourth-ventricular tuberculoma have been previously described [6,7,8,9,10,11,12,13,14]. We note that a large tuberculoma can resemble a brain tumor that compresses the surrounding brain tissue, resulting in symptoms associated with intracranial hypertension, such as headache, vomiting, and nausea [6,7,8,9,10,11,12,13,14,6,7,8,9,10,11,12,13,14,15,16,17] that were found in the present case.

The detection of CNS tuberculoma can be efficaciously achieved if the individual presenting with neurological problems has a documented bacillary history and the indicated features can be detected by computed tomography (CT) or MRI. Tuberculomas might present as a round or lobulated nodule with moderate-to-severe edema on CT. Both solid and ring appearances are typical of postcontrast enhancement results. A central calcification core with a ring of peripheral enhancement has been documented, but is not specific to TB. The lesions are generally >20 mm in diameter. In India, the most frequent radiological finding in a young adult with a new onset seizure is a solitary ring-enhancing lesion on a CT scan of the brain, with the most frequent cause being neurocysticercosis (NCC), followed by tuberculoma. MRI is the method of choice for evaluating suspected tuberculomas with a pretty strong caseous necrosis centrally on the background of a granulomatous reaction. On MRI imaging, appearance varies depending on the stage of maturation of the tuberculoma, whether noncaseating, caseating with a solid center, or caseating with a liquid center. Tuberculomas with solid caseating center appear hypo-to-isointense on both T1W and T2W images along with an iso-to-hyperintense rim on T2W images accompanied by a peripheral rim of enhancement, while tuberculomas with central liquid are hypointense on T1W images and hyperintense on T2W images along with a peripheral hypointense rim, and the rim gets enhanced on contrast study. On MRI, ring-chancing lesions such as NCC, abscess, primary brain neoplasms, and metastases can mimic the radiological features of tuberculous. MRI spectroscopy can help distinguish tuberculoma from other lesions such as NCC, lymphoma, and glioblastoma multiforme. MRI spectroscopy revealed an increased lipid peak, cholesterol ester, plasmalogen, and phenolic glycolipids, which can be used to differentiate tuberculomas from malignant tumors and other diseases. Furthermore, the presence of a lipid signal in MRI spectroscopy in the setting of a ring-enhancing lesion is highly specific for tuberculoma and has never been detected in instances of NCC [8,9,10,11,12,13,14,6,7,8,9,10,11,12,13,14,15,16,17,18,19,20].

Unfortunately, the diagnosis of CNS tuberculoma in patients without an established bacillary history can be extremely complicated [6,7,8,9,10,11,12,13,14]. Although multiple lesions have been reported in up to 33% of cases with CNS tuberculosis, the patient in this report presented with a solitary brain lesion [18]. An intracranial tuberculoma might have a differential diagnosis, such as toxoplasmosis, parasitic infection, fungal infection, high-grade glioma, and metastatic lesion [6,7,8,9,10,11,12,13,14]. Clinically and radiologically, we favored a diagnosis of primary, intra-fourth-ventricular tumor. Retrospectively, the precise diagnosis of tuberculoma, without any bacillary context, is extremely difficult, even though the MRI characteristics were able to contribute to the diagnosis, because primary, intra-fourth-ventricular tumors are more common in children.

The criterion standard for a tuberculoma diagnosis is a brain lesion biopsy, for histopathological assessment [6,7,8,9,10,11,12,13,14,6,7,8,9,10,11,12,13,14,15,16,17]. Histopathological tuberculoma evaluations always show necrotizing granulomas. Additionally, the presence of AFB within the biopsy sample should be investigated. The tissue sample should be cultured to confirm the growth of *M. tuberculosis* [4,5,6,7,8,9,10,11,12,13,14,6,7,8,9,10,11,12,13,14,15,16,17].

If a patient presents with the signs of increased intracranial pressure, the radical eradication of the lesion so as to decompress it is essential. Upon surgery, the patient may also require 18 months’ of antituberculous chemotherapy to completely eradicate the TB infection [19].

## Conclusion

Pediatric, intraventricular tuberculoma in the fourth ventricle, without systemic TB, is uncommon and extremely difficult to diagnose effectively. However, tuberculoma should be deemed as a crucially differential diagnosis for an intraventricular, ring-enhancing mass, particularly in immunocompromised individuals and patients from developing countries, to determine an appropriate treatment strategy and achieve an improved prognosis.
